# Differentiation between high-grade gliomas and solitary brain metastases: a comparison of five diffusion-weighted MRI models

**DOI:** 10.1186/s12880-020-00524-w

**Published:** 2020-11-23

**Authors:** Jiaji Mao, Weike Zeng, Qinyuan Zhang, Zehong Yang, Xu Yan, Huiting Zhang, Mengzhu Wang, Guang Yang, Minxiong Zhou, Jun Shen

**Affiliations:** 1 Department of Radiology, Sun Yat-Sen Memorial Hospital, Sun Yat-Sen University, No. 107 Yanjiang Road West, Guangzhou, 510120 China; 2 Guangdong Provincial Key Laboratory of Malignant Tumor Epigenetics and Gene Regulation, Medical Research Center, Sun Yat-Sen Memorial Hospital, Sun Yat-Sen University, No. 107 Yanjiang Road West, Guangzhou, 510120 China; 3 MR Scientific Marketing, Siemens Healthcare, No. 278 Zhouzhu Road, Shanghai, 201318 China; 4 Shanghai Key Laboratory of Magnetic Resonance, Institute of Physics and Electronics Science, East China Normal University, No. 3663 North Zhongshan Road, Shanghai, 200062 China; 5 College of Medical Imaging, Shanghai Key Laboratory of Molecular Imaging, Shanghai University of Medicine and Health Sciences, No. 279 Zhouzhu Road, Shanghai, 201318 China

**Keywords:** Glioma, Brain metastasis, Magnetic resonance imaging, Diffusion-weighted imaging, Non-Gaussian

## Abstract

**Background:**

To compare the diagnostic performance of neurite orientation dispersion and density imaging (NODDI), mean apparent propagator magnetic resonance imaging (MAP-MRI), diffusion kurtosis imaging (DKI), diffusion tensor imaging (DTI) and diffusion-weighted imaging (DWI) in distinguishing high-grade gliomas (HGGs) from solitary brain metastases (SBMs).

**Methods:**

Patients with previously untreated, histopathologically confirmed HGGs (*n* = 20) or SBMs (*n* = 21) appearing as a solitary and contrast-enhancing lesion on structural MRI were prospectively recruited to undergo diffusion-weighted MRI. DWI data were obtained using a q-space Cartesian grid sampling procedure and were processed to generate parametric maps by fitting the NODDI, MAP-MRI, DKI, DTI and DWI models. The diffusion metrics of the contrast-enhancing tumor and peritumoral edema were measured. Differences in the diffusion metrics were compared between HGGs and SBMs, followed by receiver operating characteristic (ROC) analysis and the Hanley and McNeill test to determine their diagnostic performances.

**Results:**

NODDI-based isotropic volume fraction (V_iso_) and orientation dispersion index (ODI); MAP-MRI-based mean-squared displacement (MSD) and q-space inverse variance (QIV); DKI-generated radial, mean diffusivity and fractional anisotropy (RD_k_, MD_k_ and FA_k_); and DTI-generated radial, mean diffusivity and fractional anisotropy (RD, MD and FA) of the contrast-enhancing tumor were significantly different between HGGs and SBMs (*p* < 0.05). The best single discriminative parameters of each model were V_iso_, MSD, RD_k_ and RD for NODDI, MAP-MRI, DKI and DTI, respectively. The AUC of V_iso_ (0.871) was significantly higher than that of MSD (0.736), RD_k_ (0.760) and RD (0.733) (*p* < 0.05).

**Conclusion:**

NODDI outperforms MAP-MRI, DKI, DTI and DWI in differentiating between HGGs and SBMs. NODDI-based V_iso_ has the highest performance.

## Background

High-grade gliomas (HGGs) and brain metastases are common malignancies in the central nervous system (CNS). HGGs account for approximately 80% of primary CNS malignancies [1]. Meanwhile, metastatic tumors occur ten times more frequently than primary malignancy in the brain [2]. The differentiation between HGGs and brain metastases is critical, as the management strategies for these two malignant brain tumors are vastly different. For patients with HGGs, surgical resection is the first choice, and it is usually not necessary to perform a systemic examination [2]. However, for patients with suspected brain metastases, comprehensive systemic examinations are needed, and if confirmed, stereotactic radiosurgery or systemic therapy such as targeted therapy and immunotherapy are recommended [3]*.*

Magnetic resonance imaging (MRI) is the mainstay of imaging modalities for the diagnosis of brain tumors. For patients who present multiple cerebral lesions and have a history of primary malignancy, the diagnosis of brain metastases may be straightforward by MRI. However, solitary brain metastases (SBMs) are the first manifestation in nearly 30% of patients with systemic malignancy [4]*.* Therefore, when patients show a solitary and contrast-enhancing brain lesion, it would be challenging to distinguish HGG from solitary brain metastasis (SBM) because they often show similar signal features and contrast enhancement patterns on conventional MRI, leading to incorrect diagnosis in over 40% of cases [5]. In this case, tumor biopsy is often performed to confirm the histologic diagnosis, whereas it has inherent limitations, such as procedure-related complications, interobserver variability and sampling errors [6]. Thus, a noninvasive method to differentiate HGGs from SBMs is preferable and sometimes mandatory when the patient cannot receive surgery due to poor general condition or when the tumor involves or is adjacent to important brain areas.

Diffusion-weighted imaging (DWI) is one of the most widely used advanced MRI techniques to characterize the microstructural changes in cerebral tumors, which complements the anatomic information provided by conventional MRI [7]*.* Previously, Gaussian-based DWI and diffusion tensor imaging (DTI) have been used to distinguish HGGs from SBMs, with DWI-based apparent diffusion coefficient (ADC) and DTI-generated fractional anisotropy (FA) being the most commonly used metrics. However, contradictory results have been reported on the ability of ADC and FA to differentiate HGGs from SBMs [8–10].

Recently, novel diffusion MRI techniques, such as the three-compartment biophysical model neurite orientation dispersion and density imaging (NODDI) and the non-Gaussian-based mean apparent propagator (MAP)-MRI, have emerged as powerful tools to evaluate brain microstructure in vivo, as they can provide new insights into the complexity and inhomogeneity of brain microstructure [11, 12]. Both NODDI and MAP-MRI have shown promising results in lateralization of temporal lobe epilepsy [13], assessment of Parkinson’s disease [14] and grading of gliomas [15]; nonetheless, whether they outperformed the more commonly used non-Gaussian-based DKI and Gaussian diffusion models such as DTI and DWI in differentiation between HGGs and SBMs remains unknown. Therefore, the aim of our study was to compare the diagnostic performance of NODDI, MAP-MRI, DKI, DTI and DWI in distinguishing HGGs from SBMs.

## Methods

### Study participants

**Fig. 1:**
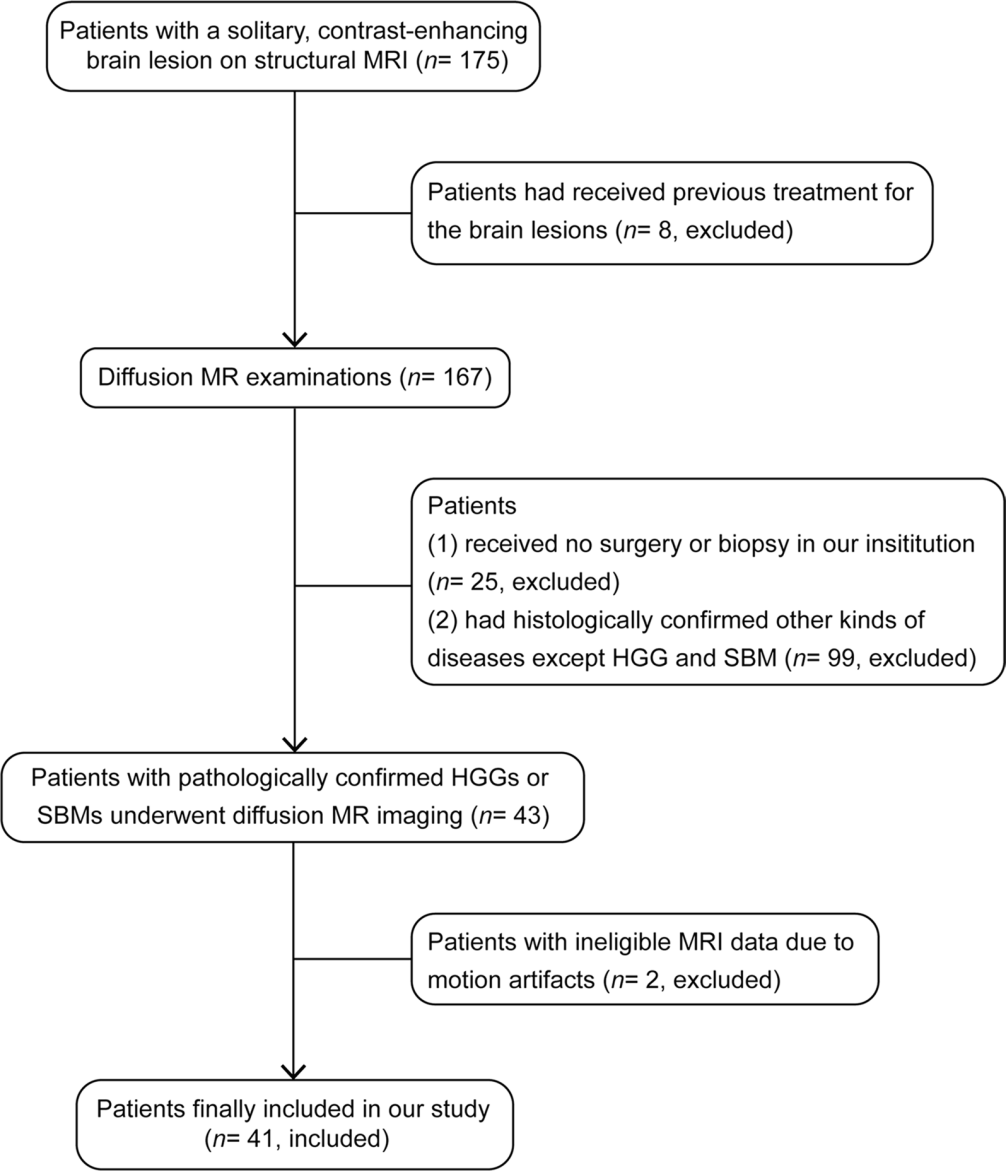
Flowchart shows the selection of the study population

Our institutional review board approved this prospective study, and all participants provided written informed consent. From January 2019 to March 2020, 175 consecutive patients who presented a solitary and contrast-enhancing brain lesion on structural MRI, which were identified by a radiologist with 3 years of experience, were enrolled to undergo diffusion-weighted MRI. The inclusion criteria were as follows: (a) a solitary and contrast-enhancing brain lesion on structural MRI, and (b) a pathological diagnosis of HGG according to the world health organization (WHO) 2016 classification of brain tumors, or a pathological diagnosis of SBM. The exclusion criteria were as follows: (a) brain lesions that had received previous treatment before MRI, (b) brain lesions that had received no surgery or biopsy after MRI, (c) histologically confirmed other diseases except HGG and SBM, and (d) poor-quality MR images due to movement artifacts. Finally, 41 patients (26 males and 15 females; mean age, 54.85 years; age, 19–81 years) were included in our study. The flowchart for the selection of the study population is shown in Fig. 1.

### MRI

All patients included in our study underwent structural and diffusion MRI on a 3.0T scanner (MAGNETOM Skyra, Siemens Healthcare, Erlangen, Germany) with a 20-channel head/neck coil. The structural MRI sequences included axial turbo spin echo (TSE) T2-weighted (T2W) imaging and axial TSE T1-weighted (T1W) imaging. After intravenous administration of 0.1 mmol/kg gadobutrol (Gadovist, Bayer Healthcare), axial contrast-enhanced TSE T1W imaging, as well as coronal and sagittal contrast-enhanced FLASH T1W imaging, was performed. On the second day after the structural MRI, diffusion MRI was performed in the axial plane using a full q-space Cartesian grid sampling procedure with a radial grid size of 3, ninety-nine diffusion directions and ten different b-values (from 0 to 3000 s/mm^2^). The acquisition time was 10 min 32 s. The acquisition parameters of all MR sequences are shown in Table 1. The geometric parameters, including slice thickness, slice gap and FOV of axial T1W and T2W, were identical to those of diffusion-weighted MRI. Moreover, the imaging planes of axial T2W and T1W and diffusion MRI were all aligned parallel to the genu and splenium line of the corpus callosum.

**Table 1:** Imaging sequences and acquisition parameters of structural and diffusion MRI

Sequence	Slice orientation	TR/TE (ms)	TI (ms)	Slice thickness/gap (mm)	FOV (mm^2^)	Matrix	Averages
TSE T2W	Axial	4500/96	–	4/0	220 × 220	320 × 240	1
TSE T1W	Axial	2000/9	900	4/0	220 × 220	320 × 240	1
FLASH T1W	Coronal	175/4.73	–	5/1	220 × 220	320 × 208	2
FLASH T1W	Sagittal	150/4.73	–	4/1	220 × 220	320 × 256	2
Diffusion MRI	Axial	6000/109	–	4/0	220 × 220	110 × 110	1

*TSE* turbo spin echo, *T2W* T2-weighted, *T1W* T1-weighted, *TR* repetition time, *TE* echo time, *TI* inversion time, *FOV* field of view

### Diffusion data analysis

All DWI data were converted to the NIfTI format using the DCM2NII tool and then processed using the NeuDiLab software developed in-house based on the open-resource tool DIPY (Diffusion Imaging in Python, http://nipy.org/dipy). The five diffusion models and the derived diffusion metrics are as follows:

For the conventional DWI model, the ADC measures the magnitude of diffusion of water molecules in a voxel, which was calculated with the following equation [16]:

$${\raise0.7ex\hbox{${S\left( b \right)}$} \!\mathord{\left/ {\vphantom {{S\left( b \right)} {S\left( 0 \right)}}}\right.\kern-\nulldelimiterspace} \!\lower0.7ex\hbox{${S\left( 0 \right)}$}} = exp\left( { - b \cdot ADC} \right)$$

where S(b) is the signal intensity according to the given b value and S (0) is the signal intensity for b = 0 s/mm^2^.

For the DTI model, the axial and radial diffusivity (AD and RD) are the average diffusivities, respectively, in the directions parallel and perpendicular to the diffusion tensor (DT) eigenvector with the largest eigenvalue. Mean diffusivity (MD) quantifies the mean extent of the diffusion of water molecules in a voxel and reflects the overall level of molecular dispersion. Fractional anisotropy (FA) represents the amount of diffusion asymmetry within a voxel, which in theory should range between 0 and 1. AD, RD, MD and FA were all derived from the principal eigenvalues (λ1, λ2, and λ3) of DT with the following equations [17]:

$$AD = \uplambda 1$$

$$RD = \frac{\uplambda 2 + \uplambda 3}{2}$$

$$MD = \frac{\uplambda 1 + \uplambda 2 + \uplambda 3}{3}$$

$$FA = {\sqrt {\frac{1}{2}}\frac{{\sqrt {\left( {\uplambda 1 - \uplambda 2} \right)^{2} + \left( {\uplambda 1 - \uplambda 3} \right)^{2} + \left( {\uplambda 2 - \uplambda 3} \right)^{2} } }}{{\sqrt {\left( {\uplambda 1^{2} + \uplambda 2^{2} + \uplambda 3^{2} } \right)} }}}$$

For the DKI model, AD, RD, MD, and FA were denoted as AD_k_, RD_k_, MD_k_, and FA_k_, respectively, which were derived from the principal eigenvalues (λ1, λ2, and λ3) of the corrected diffusion tensor (DT) with the same equations as the DTI model. The axial, radial, and mean kurtosis (AK, RK, and MK) were derived from the kurtosis tensor (KT). Specifically, AK and RK are the average kurtosis parallel and perpendicular to the principle diffusion eigenvector. MK is the average kurtosis of all diffusion directions. The DKI model is described with the following equation [18]:

$$S\left( b \right) = S\left( 0 \right)exp\left( { - bADC_{DKI} + \frac{1}{6}b^{2} \cdot ADC_{DKI}^{2} \cdot K_{ } } \right)$$

$$MK = \left( {{\raise0.7ex\hbox{$1$} \!\mathord{\left/ {\vphantom {1 n}}\right.\kern-\nulldelimiterspace} \!\lower0.7ex\hbox{$n$}}} \right)\mathop \sum \limits_{i = 1}^{n} \left( {K_{ } } \right)_{i}$$

where ADC_DKI_ is the apparent diffusion coefficient obtained with DKI and K is the kurtosis parameter.

For the MAP model, the non-Gaussianity (NG) characterizes the three-dimensional diffusion process and is defined as $NG = \sin \uptheta {\text{PG}}$, which quantifies the dissimilarity between the propagator, *P*(r), and its Gaussian part, G(r). Axial non-Gaussianity (NG_Ax_) and radial non-Gaussianity (NG_Rad_) are the derivations of NG for diffusion on the axial and radial directions, respectively. The return-to-origin probability (RTOP) describes the probability of no net displacement of molecules between two diffusion sensitization gradients, and the return-to-plane probability (RTPP) and return-to-axis probability (RTAP) are its variants for diffusion in one- and two-dimensions. The mean squared displacement (MSD) measures the average amount of diffusion in a voxel. The q-space inverse variance (QIV) measures the inverse variance of the q-signal geometric means. RTOP, RTAP, RTPP, MSD and QIV are calculated using the following equations [19]:

$$RTOP = \mathop \int \limits_{{R^{3} }}^{ } E\left( q \right)dq$$

$$RTAP = \mathop \int \limits_{{R^{2} }}^{ } E\left( {q_{ \bot } } \right)dq_{ \bot }$$

$$RTPP = \mathop \int \limits_{R}^{ } E\left( {q_{\parallel } } \right)dq_{\parallel }$$

$$MSD = \mathop \int \limits_{{R^{3} }}^{ } P\left( R \right)R^{2} d^{3} R$$

$$QIV^{ - 1} = \mathop \int \limits_{{R^{3} }}^{ } E\left( q \right)q^{2} d^{3} q$$

where q indicates the q-space wave-vector; *E*(q) is the ratio of the signal at q to that at q = 0; q_⊥_ denotes the q-vector on the sampled plane; and q_//_ denotes the component of the q-vector along the fiber axis. R denotes a three-dimensional displacement vector. *P*(R) indicates the likelihood of particles to undergo a net displacement.

For the NODDI model, the isotropic volume fraction (V_iso_) measures the isotropic diffusion compartment in a voxel, the intracellular volume fraction (V_ic_) represents diffusion within the axons and cells, and the orientation dispersion index (ODI) measures the orientation dispersion of fibers in a voxel. V_iso_, V_ic_ and ODI are calculated using the following equation [17]:

$$E = \left( {1 - V_{iso} } \right)\left( {V_{ic} E_{ic} \left( {ODI} \right) + \left( {1 - V_{ic} } \right)*E_{ec} } \right) + V_{iso} E_{iso}$$

where *E*_*ic*_ is the signal contribution from the intracellular compartment; *E*_*ec*_ is the signal due to diffusion in the extracellular space; and an isotropic Gaussian compartment *E*_*iso*_ represents free diffusion.

All diffusion parametric images and axial postcontrast T1W images were coregistered to T2W images using Elastix software (http://elastix.isi.uu.nl/) and transformed into the same imaging space. For diffusion images, baseline data with b = 0 were used for registration. Rigid transform was used due to its robustness with comparison to affine transform for the multicontrast data. For quantitative analysis, a neuroradiologist (with 8 years of experience in neuroradiology) performed region of interest (ROI) measurements with guidance from a board-certified radiologist (with 17 years of experience in neuroradiology), and both radiologists were blinded to the histological results. All the ROIs were drawn on the registered postcontrast T1W images and T2W images using the open-source application ITK-SNAP (www.itk-snap.org). The enhanced areas seen on the postcontrast T1W images were delineated and defined as the ROIs of the contrast-enhancing tumor. The hyperintense signal that represented peritumoral edema on the T2W images was manually outlined and defined as the ROI of peritumoral edema. Areas of necrosis, cysts or hemorrhage that were detectable on the postcontrast T1W images or T2W images were excluded from the ROIs. Finally, the ROIs were directly copied to the coregistered parametric diffusion maps of the same patient by using MRIcron software (https://people.cas.sc.edu/rorden/mricron/index.html) to calculate the corresponding average values of the contrast-enhancing tumor and peritumoral edema.

### Statistical analysis

The normality and equal variance of diffusion metrics were checked using the Shapiro–Wilk test and Levene’s F test, respectively. Differences in all diffusion parameters between HGGs and SBMs were compared by using the independent Student’s *t* test or the Mann–Whitney U test. The diagnostic performance of the significant diffusion metrics to discriminate HGGs from SBMs was evaluated by receiver operating characteristic (ROC) curve analysis. The optimal cutoff value, sensitivity, specificity and accuracy were calculated. The area under the curve (AUC) was compared by using the Hanley and McNeill test using the R software (version 3.2.4; R Foundation for Statistical Computing). All other statistical analyses were performed using SPSS (version 26.0; SPSS, Chicago, III, USA). A two-tailed *p* < 0.05 was considered to indicate a significant difference.

## Results

### Study population

Twenty patients (13 males and 7 females; mean age, 55.70 years; age, 19–67 years) with HGGs diagnosed by histopathology including seven patients with anaplastic astrocytoma (WHO grade III) and thirteen patients with glioblastoma (WHO grade IV) and twenty-one patients (13 males and 8 females; mean age, 54.05 years; age, 43–81 years) with SBMs confirmed by histopathology were included. The primary tumors were lung carcinoma (*n* = 10), breast carcinoma (*n* = 5), colon carcinoma (*n* = 3), liver carcinoma (*n* = 1), gastric carcinoma (*n* = 1), and thyroid carcinoma (*n* = 1).

### Diffusion parameters between HGGs and SBMs

The averages of all diffusion parameters and their comparison between the HGG group and SBM group are shown in Table 2. The RD, MD, RD_k_, MD_k_, MSD, QIV, V_iso_ and ODI of the contrast-enhancing tumors were significantly lower in the HGGs than in the SBMs (*p* = 0.006, *p* = 0.016, *p* = 0.002, *p* = 0.007, *p* = 0.002, *p* = 0.039, *p* = 0.001, and *p* = 0.003, respectively). The FA and FA_k_ of the contrast-enhancing tumors were significantly higher in the HGGs than in the SBMs (*p* = 0.007 and *p* = 0.021, respectively). No significant differences were found among all other diffusion parameters in the contrast-enhancing tumors or peritumoral edema between the two groups (*p* > 0.05).

**Table 2:** Diffusion parameters in the contrast-enhancing tumor or peritumoral edema of HGGs and SBMs

Parameters	Contrast-enhancing tumor		*p* value	Peritumoral edema		*p* value
	HGGs (*n* = 20)	SBMs (*n* = 21)		HGGs (*n* = 20)	SBMs (*n* = 21)	
DWI						
ADC (10^–3^ mm^2^/s)	0.56 ± 0.10	0.64 ± 0.14	0.068	0.57 ± 0.10	0.59 ± 0.15	0.876
DTI						
AD (10^–3^ mm^2^/s)	0.88 ± 0.11	0.93 ± 0.15	0.361	0.92 ± 0.18	0.97 ± 0.30	0.715
RD (10^–3^ mm^2^/s)	0.55 ± 0.15	0.69 ± 0.18	0.006*	0.56 ± 0.15	0.61 ± 0.26	0.754
MD (10^–3^ mm^2^/s)	0.66 ± 0.12	0.77 ± 0.17	0.016*	0.68 ± 0.15	0.73 ± 0.26	0.958
FA	0.33 ± 0.13	0.22 ± 0.09	0.007*	0.35 ± 0.11	0.33 ± 0.15	0.315
DKI						
AK	0.72 ± 0.13	0.75 ± 0.09	0.396	0.81 ± 0.12	0.74 ± 0.15	0.120
RK	0.95 ± 0.34	0.82 ± 0.19	0.137	0.95 ± 0.28	1.03 ± 0.31	0.397
MK	0.80 ± 0.20	0.78 ± 0.12	0.681	0.84 ± 0.16	0.86 ± 0.18	0.756
AD_k_ (10^–3^ mm^2^/s)	1.31 ± 0.18	1.42 ± 0.27	0.095	1.34 ± 0.24	1.44 ± 0.43	0.449
RD_k_ (10^–3^ mm^2^/s)	0.81 ± 0.21	1.09 ± 0.32	0.002*	0.83 ± 0.23	0.94 ± 0.38	0.549
MD_k_ (10^–3^ mm^2^/s)	0.97 ± 0.17	1.21 ± 0.30	0.007*	1.03 ± 0.24	1.10 ± 0.38	0.835
FA_k_	0.32 ± 0.15	0.23 ± 0.09	0.021*	0.33 ± 0.11	0.31 ± 0.13	0.309
MAP						
NG	0.27 ± 0.06	0.25 ± 0.38	0.216	0.28 ± 0.05	0.27 ± 0.05	0.426
NG_Ax_	0.22 ± 0.04	0.21 ± 0.32	0.295	0.29 ± 0.13	0.23 ± 0.44	0.192
NG_Rad_	0.15 ± 0.04	0.14 ± 0.02	0.784	0.15 ± 0.03	0.14 ± 0.03	0.359
MSD (10^−5^mm^2^)	18.77 ± 4.27	24.24 ± 6.12	0.002*	20.09 ± 4.09	21.90 ± 7.62	0.855
QIV (10^−10^mm^5^)	45.40 ± 22.28	73.67 ± 45.77	0.039*	49.77 ± 29.74	71.50 ± 95.45	0.754
RTOP (10^5^ mm^−3^)	3.28 ± 1.31	2.57 ± 0.89	0.100	3.48 ± 1.25	3.32 ± 1.34	0.691
RTAP (10^3^ mm^−2^)	5.11 ± 1.71	4.17 ± 1.17	0.068	5.41 ± 1.37	5.33 ± 1.88	0.876
RTPP (10^1^ mm^−1^)	5.03 ± 0.42	4.87 ± 0.44	0.229	5.08 ± 0.60	4.90 ± 0.68	0.404
NODDI						
V_ic_	0.49 ± 0.14	0.46 ± 0.10	0.485	0.51 ± 0.12	0.50 ± 0.15	0.796
V_iso_	0.09 ± 0.05	0.23 ± 0.12	0.001*	0.11 ± 0.07	0.16 ± 0.14	0.167
ODI	0.35 ± 0.11	0.45 ± 0.09	0.003*	0.34 ± 0.12	0.36 ± 0.14	0.631

All numerical data are presented as the mean ± standard deviation. *p < 0.05

### Diagnostic performances of diffusion metrics

**Fig. 2:**
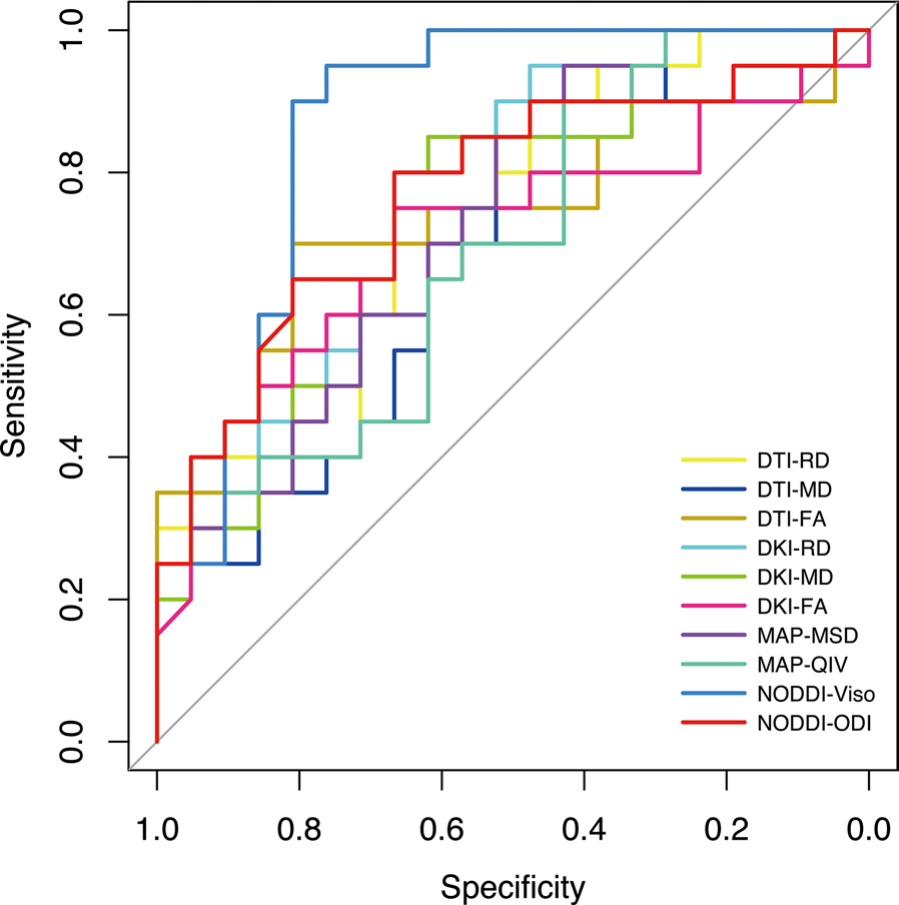
ROC curves of the significant diffusion metrics to distinguish HGGs from SBMs

**Fig. 3:**
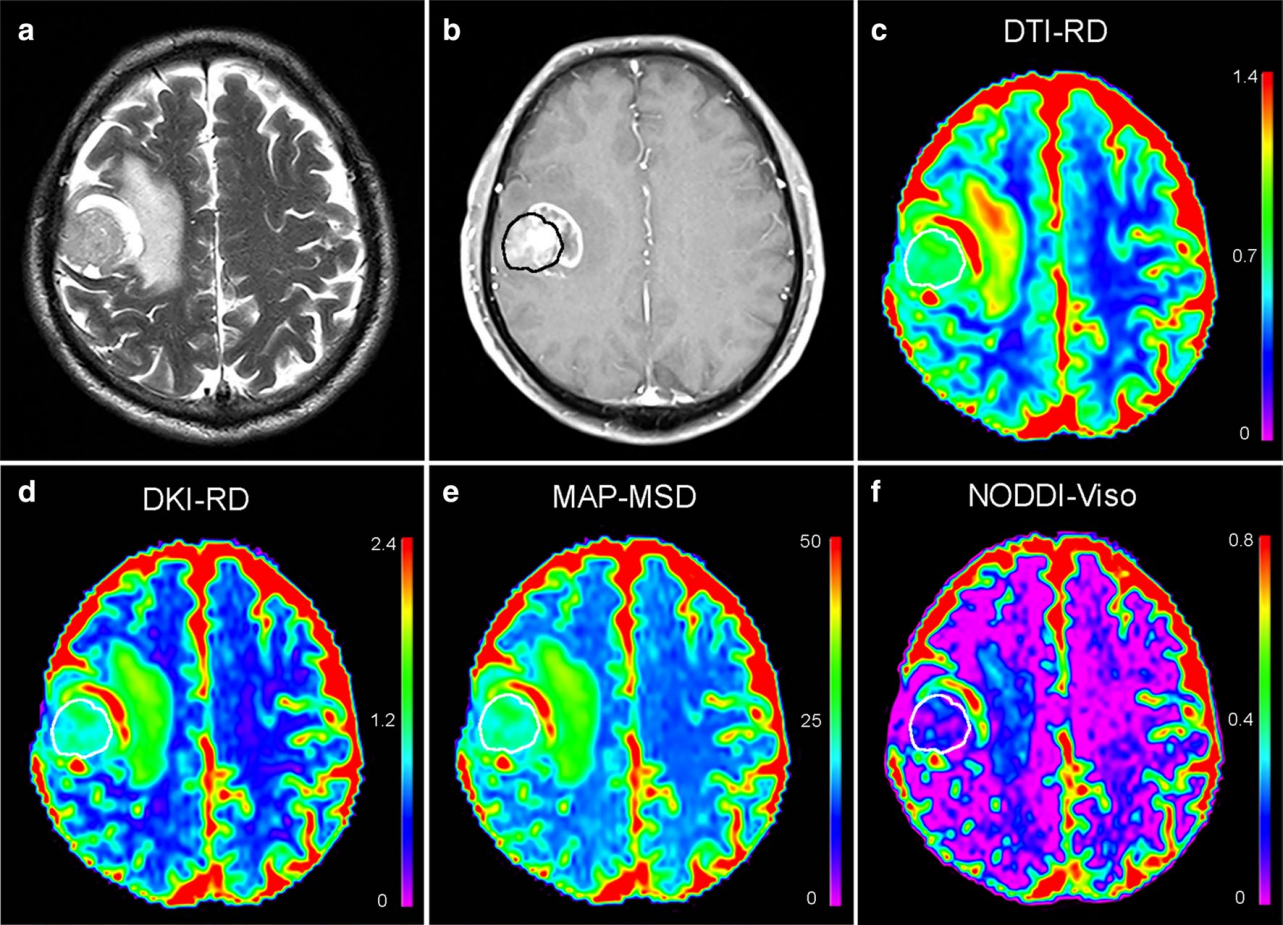
A patient with histologically confirmed glioblastoma. **a** T2W images and **b** post-contrast T1W images show a contrast-enhancing tumor with peritumoral edema located in the right frontal lobe. Pseudocolorful maps show the lesion (inside the white circle) having a slightly increased RD (**c**), RD_k_ (**d**), MSD (**e**), and V_iso_ (**f**) compared to the contralateral normal white matter

**Fig. 4:**
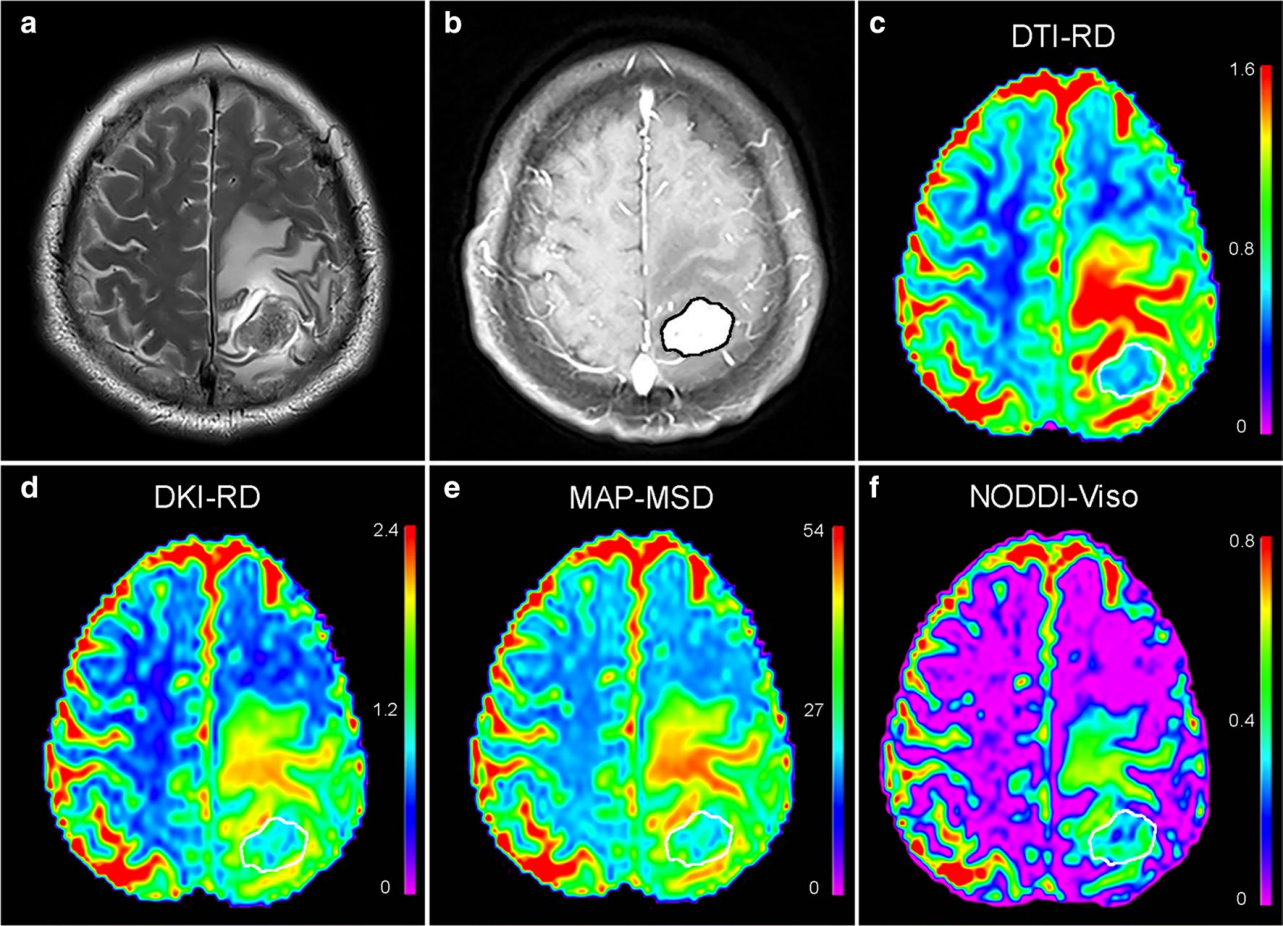
A patient with a histologically confirmed solitary brain metastasis from the colon carcinoma. **a** T2W images and **b** post-contrast T1W images represent a contrast-enhancing tumor with peritumoral edema located in the right parietal lobe. Pseudocolorful maps show the lesion (inside the white circle) having a slightly increased RD (**c**), RD_k_ (**d**), MSD (**e**) and a moderately increased V_iso_ (**f**) compared to the contralateral normal white matter

ROC curve analyses of the significant diffusion metrics of the contrast-enhancing tumors are shown in Table 3 and Fig. 2. The best single discriminative parameters for DTI, DKI, MAP-MRI and NODDI were RD, RD_k_, MSD and V_iso_, respectively (AUC = 0.733, 0.760, 0.736, and 0.871, respectively). Among them, NODDI-based V_iso_ showed the best performance in differentiating HGGs from SBMs, with a sensitivity of 95.0%, specificity of 76.2% and accuracy of 85.4% at the optimal threshold of 0.158. The AUC of NODDI-based V_iso_ was significantly higher than MAP-MRI-based MSD, DKI-based RD_k_ and DTI-based RD (*p* = 0.012; *p* = 0.047; *p* = 0.007), suggesting that NODDI outperforms MAP-MRI, DKI and DTI in distinguishing HGGs from SBMs. No significant differences were found between the AUCs of RD, RD_k_ and MSD (*p* > 0.05). Two representative cases in each group are shown in Figs. 3 and 4.

**Table 3:** ROC analyses of the significant parameters of the contrast-enhancing tumor to differentiate HGGs from SBMs

Parameters	Cutoff value	AUC (95% CI)	Sensitivity (95% CI)	Specificity (95% CI)	Accuracy (95% CI)
DTI					
RD (10^–3^ mm^2^/s)	0.597	0.733(0.580, 0.887)	0.700(0.457, 0.872)	0.667(0.431, 0.845)	0.683(0.519, 0.819)
MD (10^–3^ mm^2^/s)	0.797	0.686(0.522, 0.850)	0.900(0.669, 0.982)	0.428(0.226, 0.656)	0.658(0.494, 0.799)
FA	0.287	0.731(0.567, 0.894)	0.700(0.457, 0.872)	0.810(0.574, 0.937)	0.756(0.597, 0.876)
DKI					
RD_k_ (10^–3^ mm^2^/s)	1.106	0.760(0.613, 0.906)	0.950(0.730, 0.997)	0.476(0.264, 0.697)	0.707(0.545, 0.839)
MD_k_ (10^–3^ mm^2^/s)	1.084	0.745(0.593, 0.898)	0.850(0.611, 0.960)	0.619(0.387, 0.810)	0.732(0.571, 0.858)
FA_k_	0.255	0.711(0.544, 0.877)	0.750(0.506, 0.904)	0.667(0.431, 0.845)	0.707(0.545, 0.839)
MAP					
MSD (10^−5^mm^2^)	24.688	0.736(0.683, 0.888)	0.950(0.730, 0.997)	0.428(0.226, 0.656)	0.683(0.519, 0.819)
QIV (10^−10^mm^5^)	75.891	0.688(0.524, 0.852)	0.900(0.669, 0.982)	0.428(0.226, 0.656)	0.658(0.494, 0.799)
NODDI					
V_iso_	0.158	0.871(0.756, 0.987)	0.950(0.730, 0.997)	0.762(0.524, 0.909)	0.854(0.708, 0.944)
ODI	0.409	0.770(0.621, 0.919)	0.800(0.557, 0.934)	0.667(0.431, 0.845)	0.732(0.571, 0.858)

*CI* confidence interval

## Discussion

Our results demonstrated that HGGs and SBMs showed distinctive NODDI, MAP-MRI, DKI and DTI-based diffusion metrics in the contrast-enhancing tumor region, while no difference was observed for any of the diffusion parameters in peritumoral edema. NODDI-based tumoral V_iso_ had the greatest discriminative power between HGGs and SBMs.

Patients with HGGs or SBMs generally have a dismal prognosis, but correct differential diagnosis and appropriate clinical decisions can significantly prolong the survival time [20]. Thus, it is crucial to distinguish HGGs from SBMs. Nonetheless, it is always challenging to differentiate between these two malignancies only by conventional MRI [5]. In recent years, advanced diffusion-weighted MRI techniques have emerged as powerful tools to assess microstructural changes in CNS diseases. As the complex microstructures in neural tissue (e.g., cell membranes and myelin fibers) change water molecule diffusion into a non-Gaussian probability distribution, non-Gaussian diffusion models such as MAP-MRI and DKI are supposed to reflect the real situation of water molecule diffusion more accurately and better characterize the complexity and inhomogeneity of the tissue microenvironment than Gaussian diffusion models [21]. Specifically, DKI is a commonly used and moderately complex physical model that is sensitive to DWI sampling and noise [22]. MAP-MRI is a more recent and highly complex physical model that can evaluate three-dimensional q-space data [11] but shows increased sensitivity to DWI sampling and noise [22]. Comparatively, NODDI is an increasingly popular biophysical model with low complexity that attempts to separate the signal contribution of neural tissue into three compartments, including restricted, hindered, and isotropic diffusion, and to model the dispersion of axonal fibers [17]. NODDI metrics are highly stable to DWI sampling and image quality [22]. In the present study, we found that NODDI-based V_iso_ outperformed other non-Gaussian or Gaussian diffusion parameters in the differentiation between HGGs and SBMs. Although no single diffusion parameter can fully capture the complexity of neural tissue, our findings suggest that NODDI-V_iso_ could potentially be a sensitive imaging biomarker in neuro-oncology research and deserves further investigation.

NODDI-V_iso_ represents isotropic diffusion within the tissue; in our study, HGGs showed a lower tumoral V_iso_ value than SBMs. This phenomenon can be explained by the fact that HGGs are characterized by enlarged extracellular space and overproduction of certain components of extracellular matrix components, mainly tenascin [23]. These molecules accumulate and orient in the extracellular matrix [24], resulting in less isotropy at DWI. In contrast, metastatic brain tumors degrade the extracellular matrix with heparanase and matrix metalloproteinases, thereby growing into the brain parenchyma in an expansive and noninfiltrating pattern [25], resulting in higher isotropy at DWI. NODDI-based ODI represents the orientation dispersion of fibers in tissue [12]. In this study, the tumoral ODI value was found to be lower in HGGs than in SBMs, which could also be explained by the fact that tumor tissue tends to be less isotropic for HGGs than for SBMs. For MAP-MRI, we found that HGGs showed lower MSD and QIV values than SBMs. MSD indicates the mean square displacement of the water molecules. These results can be explained by the fact that the solid part of HGGs had higher cellularity than did brain metastases [26–28], which might lead to higher diffusion restriction in HGGs, and the water molecules will thus move shorter distances and result in a lower MSD value [19]. QIV signifies the q-space inverse variance, which is a pseudodiffusivity measure and represents different diffusion components [29]; thus, a higher tumoral QIV value for SBM suggested a higher proportion of fast diffusivity in SBMs. For DTI, we found that HGGs showed a higher tumoral FA value and lower tumoral RD and MD values than SBMs. FA is a measurement of the directionality of water diffusion along with the white matter, which has a positive correlation with tumor cellularity [30]. Higher tumoral FA values for HGGs than SBMs were also described in recent studies [31, 32], where a higher FA value of the contrast-enhancing region of HGGs was reported to be assumed to be due to the higher cellularity of HGGs [26–28]. MD reveals the rate of water molecule diffusional motion; RD represents the diffusion rate of water perpendicular to white matter fibers [17]. Both MD and RD show an inverse relationship with tumor cellularity [33, 34], which can explain the opposite change trend of MD and RD compared with FA. As an extension of DTI [35], DKI-based RD, MD and FA in our study showed similar change patterns with DTI-based RD, MD and FA.

Previously, the ability of diffusion MR metrics such as ADC and FA to distinguish peritumoral edema of HGGs from that of SBMs has been widely investigated, but the study results remain controversial [8–10]. In the present study, although five diffusion models were utilized, no significant differences were found in any of the diffusion parameters between the peritumoral edema of HGGs and SBMs. These inconsistent results may contribute to the intrinsic heterogeneity of HGGs. Although it was confirmed histologically that tumor cells exist in the peritumoral edema of HGGs, the magnitude of tumor cell infiltration actually has a substantially wide range [36]. Thus, minimal tumor infiltration may not cause a significant signal change in diffusion MRI. Further studies applying three-dimensional texture analysis of volumetric diffusion MR images could provide additional information on the heterogeneity of tumor cell infiltration in peritumoral edema, which may be helpful in the differentiation of tumor-infiltrated edema from purely vasogenic edema.

Our study has some limitations. First, the sample size was small for both the HGG and SBM groups, as all patients were prospectively enrolled from a single institution. However, our study showed that the advanced diffusion-weighted technique NODDI-based V_iso_ had a desirable diagnostic performance (AUC = 0.871) for distinguishing HGGs from SBMs. This model deserves further study with a larger sample size to validate the current results. Second, the diffusion MR examination in our study requires a long scan duration of approximately 10 min. In the future, this problem can be overcome by using advanced techniques, such as compressed sensing [37] and simultaneous multislice acquisition techniques [38].

## Conclusion

Our study shows that NODDI outperforms MAP-MRI, DKI, DTI and DWI in distinguishing HGGs from SBMs. Among all the diffusion metrics, NODDI-based V_iso_ has the highest performance in differentiating between HGGs and SBMs.

## Data Availability

The datasets used or analyzed during the current study are available from the corresponding author on reasonable request.

## Abbreviations

NODDI: Neurite orientation dispersion and density
MAP: Mean apparent propagator
MRI: Magnetic resonance imaging
DKI: Diffusion kurtosis imaging
DTI: Diffusion tensor imaging
DWI: Diffusion-weighted imaging
HGG: High-grade glioma
SBM: Solitary brain metastasis
ROC: Receiver operating characteristic
V_iso_: Isotropic volume fraction
ODI: Orientation dispersion index
MSD: Mean-squared displacement
QIV: Q-space inverse variance
RD_k_: DKI-generated radial diffusivity
MD_k_: DKI-generated mean diffusivity
FA_k_: DKI-generated fractional anisotropy
RD: Radial diffusivity
MD: Mean diffusivity
FA: Fractional anisotropy
CNS: Central nervous system
ADC: Apparent diffusion coefficient
WHO: World Health Organization
TSE: Turbo spin echo
T2W: T2-weighted
T1W: T1-weighted
AD: Axial diffusivity
DT: Diffusion tensor
AD_k_: DKI-generated axial diffusivity
AK: Axial kurtosis
RK: Radial kurtosis
MK: Mean kurtosis
KT: Kurtosis tensor
NG: Non-Gaussianity
NG_Ax_: Axial non-Gaussianity
NG_Rad_: Radial non-Gaussianity
RTOP: Return-to-origin probability
RTPP: Return-to-plane probability
RTAP: Return-to-axis probability
V_ic_: Intracellular volume fraction
ROI: Region of interest
AUC: Area under the curve
